# Isolated Trapeziometacarpal Joint Dislocation With Five Years of Follow-up: A Case Report and Review of the Literature

**DOI:** 10.7759/cureus.34631

**Published:** 2023-02-04

**Authors:** Zied Missaoui, Mohamad K Moussa, Mohammad O Boushnak, Ahmad A Abed Ali, Ali H Alayane

**Affiliations:** 1 Orthopedic Surgery, Grand Hôpital de l'Est Francilien - site de Meaux, Meaux, FRA; 2 Orthopedic Surgery, Lebanese University, Faculty of Medical Sciences, Beirut, LBN; 3 Orthopedics and Traumatology, Sunshine Coast Orthopedic Group, Sunshine Coast, AUS

**Keywords:** cmc joint dislocation, isolated dislocation, hand surgery, carpometacarpal joint dislocation, trapezio-metacarpal joint dislocation

## Abstract

Isolated trapezio-metacarpal joint dislocation is a rare injury. Despite being simple to reduce, there is not yet a consensus regarding how to secure the reduction, the type of immobilization, and the postoperative protocol. Herein, we present a rare case of pure trapezio-metacarpal joint dislocation without any associated fractures that was treated with closed reduction and intermetacarpal fixation, six weeks of immobilization, and an early rehabilitation protocol.

## Introduction

Dislocation of the trapezio-metacarpal joint (TMJ) is a rare injury that accounts for only 1% of all hand injuries. Most of these dislocations occur with associated fractures such as Bennett fractures. However, pure dislocations without associated fractures are rare; as a result, the ways to manage these injuries are not well described in the literature. Most related articles are case reports and case series of low evidence [1-3]. The mechanism of this injury is usually axial compression of the thumb in the flexed position causing a posteriorly directed dislocation [4].

The ligamentous stability of the TMJ has been subject to huge debates and controversies in order to try to find the key stabilizing ligament of this joint. The aim of these debates was to accurately target the ligamentous problems in the treatment protocol, hence achieving optimal results [5,6]. That is why treatment options are varied, ranging from closed reduction and immobilization to surgical reduction and fixation with or without soft tissue procedures [7-8].

## Case presentation

**Figure 1:**
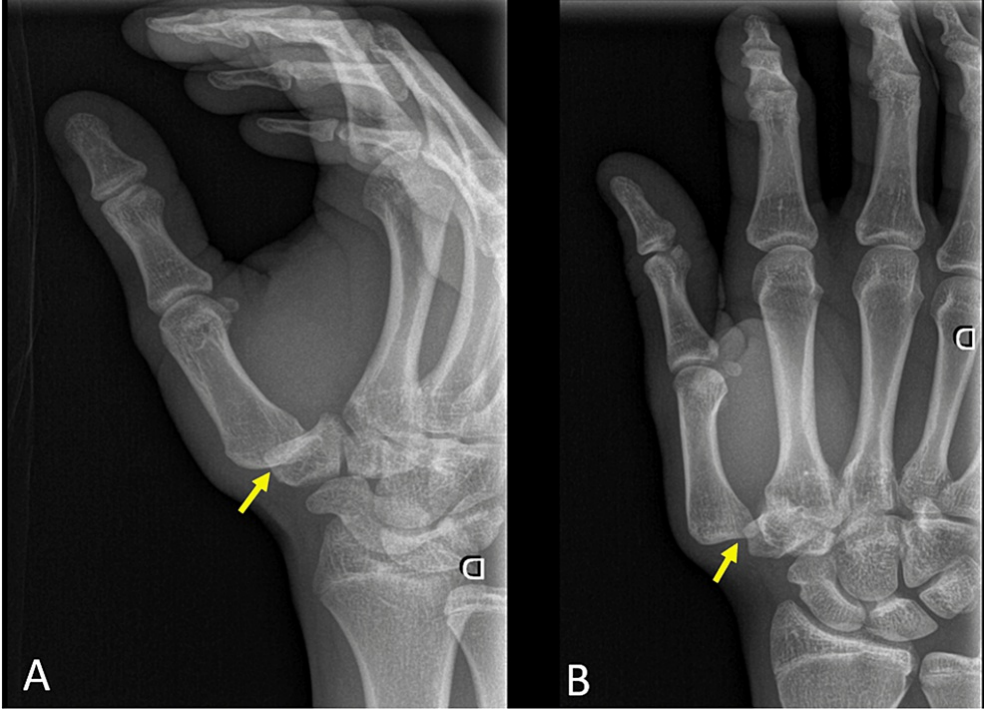
(A, B) Anteroposterior and oblique radiographs of the right hand showing isolated dislocation of the right TMJ without any associated fractures.

An 18-year-old male patient presented to our hospital after sustaining a motor vehicle accident and landing on both hands. Physical examination was relevant for edema and tenderness over the left wrist and right first metacarpophalangeal joint along with deformation of the thumb. Radiographs showed the left distal radius fracture and isolated dislocation of the right TMJ without any associated local fractures (Figure 1).

**Figure 2:**
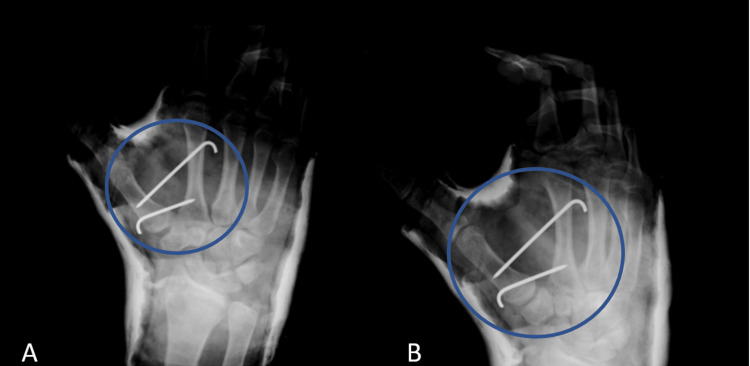
(A, B) Post operative radiographs of the hand showing a reduced trapezio-metacarpal joint that is fixed using Iselin extra-focal pinning technique.

He was then scheduled for surgical reduction and fixation. Under general anesthesia, closed reduction was easily achieved, and intermetacarpal Kirshner (K)-wires (Iselin technique) were used to secure the reduction and allow ligamentous healing to occur with time and stabilize the joint. Postoperative radiographs are shown in Figure 2.

**Figure 3:**
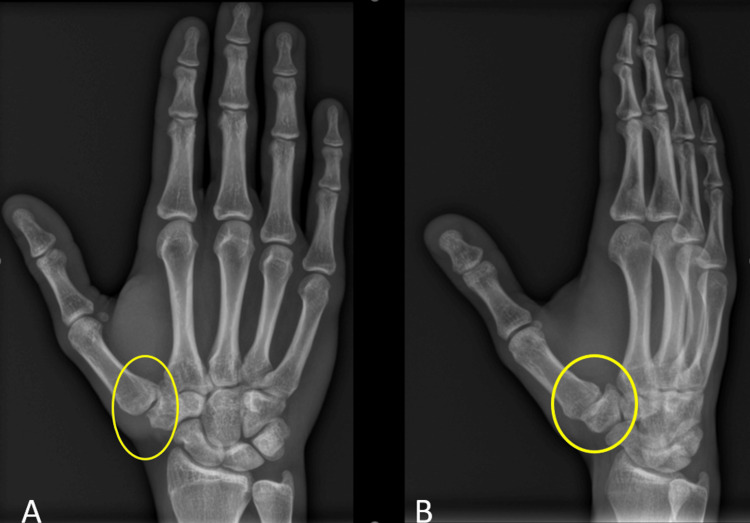
(A, B) Anteroposterior and oblique radiographs of the right hand three months postoperatively.

After six weeks of immobilization with a cast, the K-wires were removed. Testing of the TMJ in the operating room during the removal procedure showed a stable TMJ with a complete absence of laxity. Physiotherapy was started directly after the operation. After the removal of the K-wires, the patient achieved substantial improvement functionally, his disabilities of the arm, shoulder, and hand (DASH) score was 7.5/100. Two months postoperatively, the DASH score improved even more to 0.8/100. He resumed his work two months postoperatively and was allowed to return to his sports activities three months from the time of injury. Radiographs at three months are shown in Figure 3.

**Figure 4:**
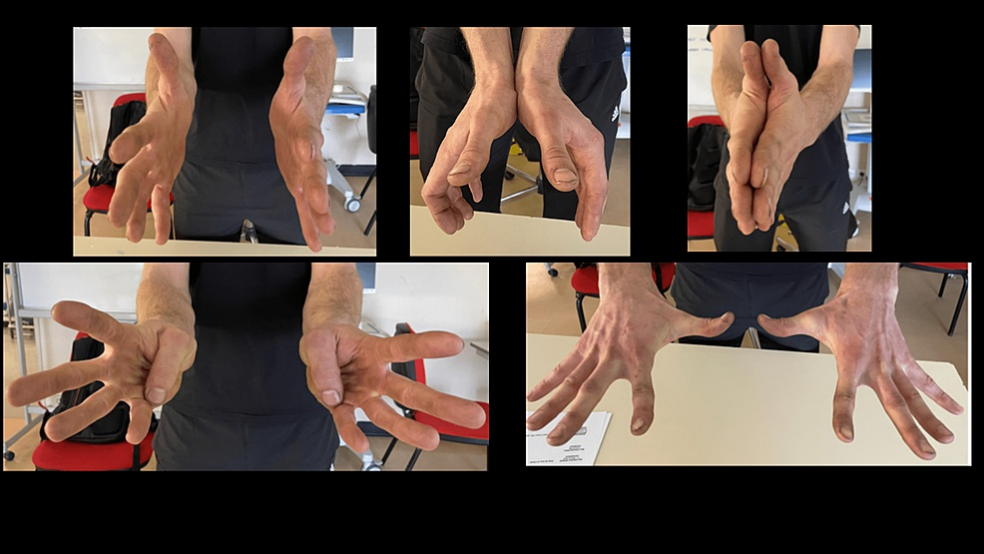
Clinical photographs of the hands at the five-year follow-up showing full range of motion of the thumb without residual deformity.

At five years postoperatively, the patient was symptom-free, and his DASH score was 0/100. He was fully functional without any stiffness, restriction in range of motion, or residual pain as shown in Figure 4.

**Figure 5:**
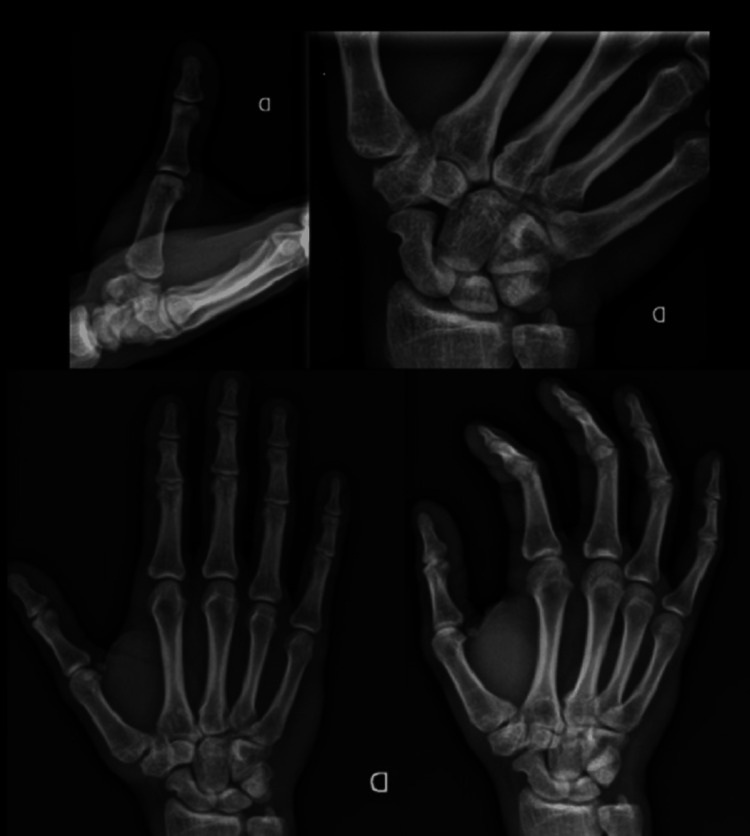
Radiographs with different views of the right hand at the five-year follow-up showing minimal degenerative changes of the first trapezio-metacarpal joint.

Radiographs of the hand did not show any signs of osteoarthritis (Figure 5).

## Discussion

TMJ dislocation accounts for less than 1% of all hand injuries. Most of these injuries fall in the category of fracture-dislocation variant injury. Pure TMJ without any fracture remains a very rare injury that is not well illustrated in literature [1-3].

Understanding the ligamentous anatomy of TMJ helps in comprehending the epidemiological difference, as well as the pathophysiology of these injuries, thereby leading to the adoption of better treatment strategies. The TMJ is stabilized by four ligamentous structures which are the anterior oblique ligament, the dorsoradial ligament, the posterior oblique ligament, and the intermetacarpal ligaments [9].

The strong volar ligament facilitates avulsion-type fracture and explains in part the rarity of pure TMJ dislocations. This ligament was previously thought to be the key stabilizer of the TMJ [9]. This was debated by Harvey et al. in 1976 and Pagalidis et al. in 1981, who demonstrated that intermetacarpal ligaments and the posterior oblique ligament were the most substantial contributors to joint stability [10,11].

On the other hand, dorsal stability is provided by a thin dorsal capsule and the strong dorsoradial ligament complex which represents the primary restraint for dorsal dislocations. The rupture of this complex facilitates dorsal dislocation. This was demonstrated by a large cadaveric study undergone by Strauch et al. in 1994 who concluded that axial loading to the thumb with associated flexion will dislocate the metacarpal bone dorsally after rupture of the thin capsule along with the dorsoradial ligament. They defined a spectrum of ligamentous injury, ranging from rupture of dorsoradial ligament with stripping of the distal volar ligament to complete avulsion of all the ligaments [12].

Diagnosis is usually made with standard radiographs of the hand. The sensitivity of the exam can be increased by applying abduction stress to the thumb. This is particularly important in subtle dislocation or subluxation which can be frequently missed leading to late diagnosis and chronic instability [13].

Treatment of TMJ dislocation involves the reduction of the dislocation, followed by fixation to secure reduction. The choice of fixation options ranges from simple cast to percutaneous pinning, to open capsulorrhaphy and ligamentous reconstruction. Because of the rarity of this condition, there is a scarcity of comparative studies between different treatment options [9]. This choice is still the subject of debate.

In 1987, Watt et al. published a series of 12 patients; they compared the results of closed reduction and six-weeks-cast-immobilization with closed reduction percutaneous pinning and cast immobilization. The best results were found in patients where percutaneous pinning was utilized for stabilizing the dislocation (two patients had no pain and no residual instability, and one patient had residual asymptomatic subluxation) [14]. Many authors adopted this method of immobilization with satisfactory results when closed reduction was combined with percutaneous pinning [9].

However, in 1988, Jakobsen et al. performed closed reduction and percutaneous pinning fixation on their 41-year-old patient. They had satisfactory good short-term outcomes followed by residual instability at the 18-month follow-up. The authors concluded that TMJ dislocation must be considered a highly unstable lesion that necessitates open reduction and ligamentous reconstructions [13]. This recommendation was adopted by Chen et al., who published the first result of open ligamentous reconstruction for the treatment of TMJ dislocation in one patient with good results [3].

In a later study, Simonian et al. (1996) compared the results of pinning versus open ligamentous reconstruction on a series of 13 patients. They had more instability in the pinning group and more osteoarthritis in the ligamentous reconstruction group [4]. And again, no evident conclusion can be drawn from this study due to its low evidence.

Some authors recommend testing the joint stability at each step of the intervention in order to decide if ligamentous reconstruction is needed or not [15]. Ligament reconstruction remains a mandatory valid option in cases of neglected chronic TMJ dislocation. It may also be a reasonable choice in cases of young athletic patients and manual workers [15,16].

## Conclusions

Pure TMJ dislocation remains a rare hand injury that deserves special attention when planning for the best treatment option. Reduction is usually easily achieved but the choice of fixation is still debatable. Simple immobilization by cast should be avoided due to the lack of supportive satisfactory results in the literature. Percutaneous pinning and ligamentous reconstruction remain the best fixation modality. Further studies may be required to confirm the superiority of one stabilization method over another.
